# Modelling tooth–prey interactions in sharks: the importance of dynamic testing

**DOI:** 10.1098/rsos.160141

**Published:** 2016-08-10

**Authors:** Katherine A. Corn, Stacy C. Farina, Jeffrey Brash, Adam P. Summers

**Affiliations:** 1 Department of Ecology and Evolutionary Biology, Cornell University, Ithaca, NY, USA; 2 Museum of Comparative Zoology, Harvard University, Cambridge, MA, USA; 3 Valley Steel and Stone, Friday Harbor, WA, USA; 4 School of Aquatic and Fisheries Sciences, University of Washington, Friday Harbor, WA, USA

**Keywords:** saw, cutting performance, *Hexanchus griseus*, *Galeocerdo cuvier*, *Carcharhinus*

## Abstract

The shape of shark teeth varies among species, but traditional testing protocols have revealed no predictive relationship between shark tooth morphology and performance. We developed a dynamic testing device to quantify cutting performance of teeth. We mimicked head-shaking behaviour in feeding large sharks by attaching teeth to the blade of a reciprocating power saw fixed in a custom-built frame. We tested three tooth types at biologically relevant speeds and found differences in tooth cutting ability and wear. Teeth from the bluntnose sixgill (*Hexanchus griseus*) showed poor cutting ability compared with tiger (*Galeocerdo cuvier*), sandbar (*Carcharhinus plumbeus*) and silky (*C. falciformis*) sharks, but they also showed no wear with repeated use. Some shark teeth are very sharp at the expense of quickly dulling, while others are less sharp but dull more slowly. This demonstrates that dynamic testing is vital to understanding the performance of shark teeth.

## 1. Introduction

**Figure 1.**
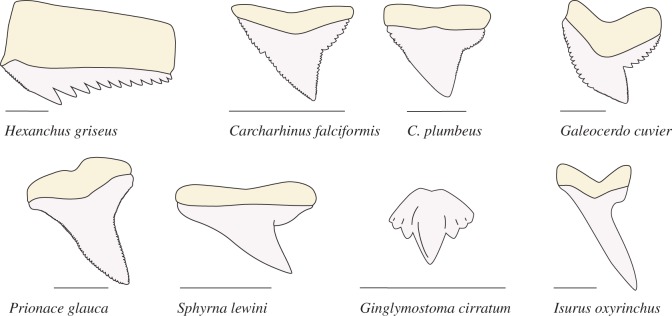
Morphological diversity in shark teeth. Shark teeth exhibit a high degree of morphological variation. These line drawings of eight tooth types demonstrate diversity in tooth shape. Scale bar, 1 cm.

Conventional wisdom suggests that mammals have the greatest diversity in tooth morphology, but another vertebrate group rivals them—sharks. Shark tooth shape is so distinctive that a single tooth can often be attributed to a particular species, especially among large predatory sharks. Though some teeth are simple triangles (e.g. silky shark teeth, *Carcharhinus falciformis*), many are spear shaped (e.g. mako shark teeth, *Isurus oxyrinchus*), deeply notched (e.g. tiger shark teeth, *Galeocerdo cuvier*) or multi-cusped (e.g. sixgill shark teeth, *Hexanchus griseus*) (figure 1). But unlike in mammals, the relationship between tooth morphology and function is not readily discernable. Despite their variation, teeth from several species of shark performed equally well in tests of puncture and unidirectional draw force of individual teeth through prey tissue [1]. There are also few theoretical predictions about cutting performance that can be drawn from morphology alone [2]. Perhaps the variation in tooth shape across large sharks reflects selection for dynamic tooth–prey tissue interactions, which is not easily discernable through morphological study and static testing regimes.

Biological materials are nearly all viscoelastic, which means loading rate affects the response to load. Typically, at high strain rates, a viscoelastic prey item will be stiffer, stronger and more brittle than at lower strain rates. Most large, predatory sharks feed with an open-mouthed strike, followed by vigorous and rapid head-shaking to cut through tissue with their teeth [3–5]. The dynamic nature of shark predation raises the possibility that differences in tooth performance will not be clear unless the speed of the interaction can be imitated. For example, ballistic testing of crossbow arrows recently showed that penetration performance depends heavily on the kinetic energy of arrows when fired into ballistic gelatin [6]. We designed a testing system to quantify performance of shark teeth as cutting implements under biologically relevant dynamic testing conditions. Our design goals were to: (i) allow multiple teeth to simultaneously interact with prey, (ii) cut bi-directionally (forward and backward) to mimic head shaking, and (iii) permit measurement of amount of prey tissue cut per tooth.

## 2. Material and methods

**Figure 2.**
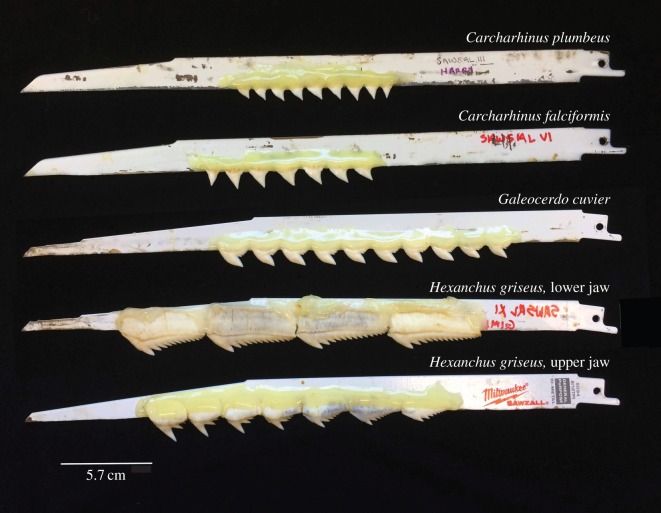
Example blades. Teeth were attached to blades using epoxy, with the lingual face against the blade. We used separate blades for teeth from the upper and lower jaw of *Hexanchus griseus*.

Test species were chosen to represent three distinct tooth morphologies (figure 2): triangular and pointed with small serrations across the edges (silky shark, *C. falciformis* and sandbar shark, *C. plumbeus*), triangular and pointed with large serrations and a deep posterior notch (tiger shark, *G. cuvier*), and elongate teeth with a series of cusps (sixgill shark, *H. griseus*). Teeth were removed from dried or frozen jaws, or obtained as loose teeth. All teeth were in excellent condition with no visible damage or wear, and only teeth from the first or second tooth rows within the jaw were used. In *G. cuvier* and *H. griseus*, teeth from both upper and lower jaws were used. We used separate blades for the upper jaw and the lower jaw teeth of *H. griseus*. For *Carcharhinus* species, only upper jaw teeth were used, as the lower jaw teeth are specialized for puncture.

Teeth were attached to 12^″^ Bi-Metal blades (Ace Hardware and Milwaukee) after the metal teeth on the blades were ground to a flat surface with a bench grinder. Quick-setting epoxy (Ace Hardware) adhered teeth to the blades and was cured for a minimum of 24 h in a warm, dehumidified room. The teeth were placed with the lingual side of the root abutting the metal blade, and epoxy was applied so that it covered the labial portion of the root. Teeth were arranged end-to-end until they covered a minimum of 25% of the blade, ensuring that teeth would be in contact with the prey during the majority of the excursion of the saw. For each tooth type (*G. cuvier*, *H. griseus* and *Carcharhinus*), three blades were tested in five successive trials, and one blade from each type was tested over 18 successive trials to examine wear patterns. We combined *C. falciformis* and *C. plumbeus* into one tooth type, because differences in tooth morphology between the species are slight and probably do not have significant functional consequences.

**Figure 3.**
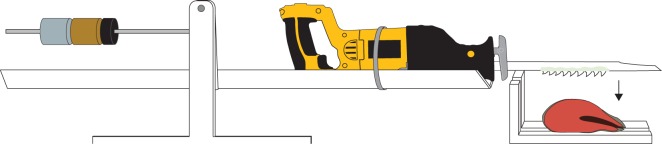
Sawing apparatus with fulcrum. Blades were used on a reciprocating saw mounted on a fulcrum. Depression and release of the left side of the fulcrum allowed the right side, holding the saw, to fall onto the section of salmon and through a gap in the cutting board.

A reciprocating saw (DeWalt model 385) was affixed to a fulcrum made of mild steel, stainless steel, and brass that allowed vertical articulation with little friction and near constant load over the normal range of motion (figure 3). Depression and release of the left side of the fulcrum allowed the right side, holding the saw, to drop onto the prey. The blade was held between 2 and 4 cm above the prey and allowed to fall onto the prey with release of the fulcrum (electronic supplemental material, Video 1). A gap in the holding plate allowed the saw to pass fully through the prey. The speed of the saw was kept within a limited range, between 2800 and 2900 reciprocations min^−1^. The blade travelled 5.715 cm per reciprocation, producing a speed of 2.67 to 2.76 m s^−1^. We estimated velocity of head shaking in sharks using 11 publically available videos from the website YouTube.com. Head shaking rates were between 0.75 and 5.75 cycles s^−1^. Assuming a range of 10–50 cm travelled per hemicycle, tooth velocity is 0.15–5.5 m s^−1^, and our tooth testing speed fell in the middle of this range. We used fresh frozen Alaskan chum salmon (*Oncorhynchus keta*) as prey, with the head and viscera removed, thawed to 23°C. The force with which the blade first contacted the prey was kept constant by counter weights (figure 3). We used a force gauge (Imada DS-50) to determine that the blade applied approximately 20 N of force, which is less than 10% of the force expected during biting by a 1.5 m shark [7].

We recorded trials using high-speed video (Casio Exilim EX-FH20) at 420 frames s^−1^ (fps) and regular video (Sony MHS-TS10) at 30 frames s^−1^. Photos were taken before and after each cut. ImageJ (using Fiji) v. 2.0.0 [8] was used to measure the amount of prey cut per reciprocation. For each trial, we calculated ‘tooth cutting ability’ by determining the contribution of a single tooth to vertical displacement of the blade through prey with the following equation:

2.1 $$\text{tooth}\;\text{cutting}\;\text{ability}=\frac{\Delta y}{6wl^{-1}}$$

where Δ*y* is the vertical displacement of the blade as it cuts through the prey, *w* is the average width of a single tooth from end to end and *l* is the length of a single stroke (5.715 cm). The denominator is multiplied by six, because vertical displacement was measured during the first six reciprocations of the saw for all trials. A higher cutting ability score indicates better performance. We compared tooth performance with an ANOVA on cutting ability for three blades of each tooth type using the *stats* package in R [9]. To control for wear, we only calculated cutting ability during the first six reciprocations of the saw once it was in contact with the prey. We quantified tooth wear from one blade for *H. griseus, G. cuvier* and one of the two *Carcharhinus* species (*C. falciformis*) by plotting cutting ability during the first six reciprocations of each trial over the course of 17 successive trials. We did not include the first cut of *C. falciformis*, because the blade bounced considerably upon first contact with the prey, introducing substantial error into measurement of vertical blade displacement. We pooled upper and lower jaw blades for teeth from *H. griseus,* because, although some teeth are smaller in the upper jaw of *H. griseus* than teeth of the lower jaw, we do not feel that the teeth were different enough to warrant separate treatment. Data are publically available at the Dryad Digital Repository (doi:10.5061/dryad.92j80) [10].

## 3. Results

**Figure 4.**
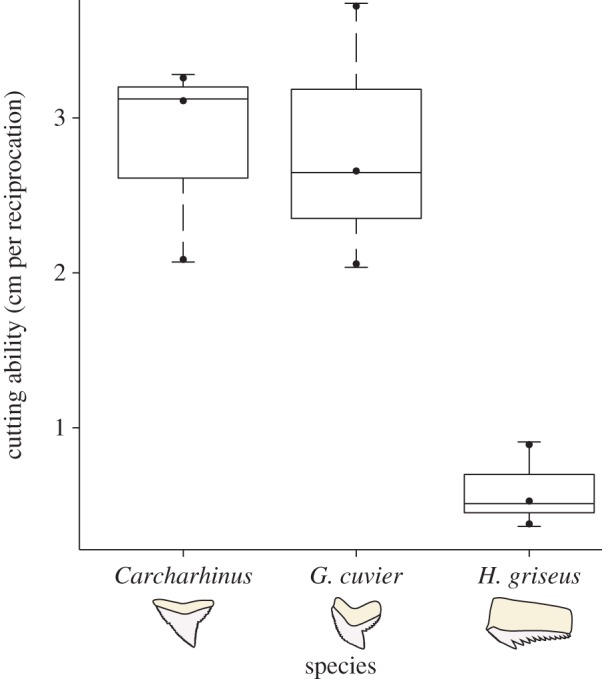
ANOVA results from cutting tests. ANOVA revealed performance differences in cutting ability among tooth morphologies (*n* = 3 for each species; d.f. = 2, 6; *p* < 0.01). Post hoc testing showed that *Hexanchus griseus* teeth had lower cutting ability than the *Galeocerdo cuvier* and *Carcharhinus* tooth types.

**Figure 5.**
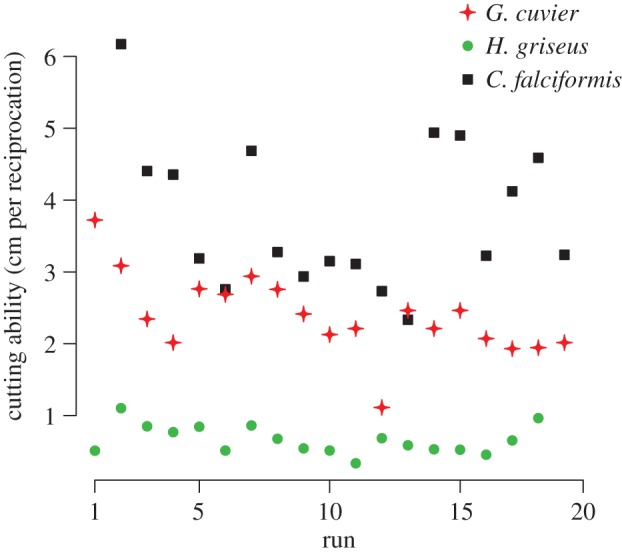
Successive testing results. Repeated use of a single blade over 17–19 trials resulted in a general pattern of decreased cutting ability in *Galeocerdo cuvier* and *Carcharhinus falciformis*.

The ANOVA (figure 4) showed significant differences in cutting ability across tooth morphologies (*F* = 12.47; *p* = 0.0073; d.f. = 2, 6). Post hoc analysis with a Tukey's honest significant differences test revealed no difference between *Carcharhinus* and *G. cuvier*, but *H. griseus* teeth had a lower cutting ability than the other two species. Teeth from the upper and lower jaw of *H. griseus* performed similarly (lower jaw: 0.39 cm^−1^–0.51 cm^−1^; upper jaw: 0.89 cm^−1^). *Carcharhinus falciformis* teeth demonstrated large net wear (decrease in cutting ability) over 17 cuts, despite showing large variation in cutting performance. *Galeocerdo cuvier* teeth showed rapid wear, with most of the cutting ability lost over the first five cuts. *Hexanchus griseus* teeth performed variably, with no pattern of wear (figure 5). Over the course of our experiments, four teeth chipped on separate blades, on either the first or second trial; all four were in the central third of the blades. The epoxy did not fail during testing.

## 4. Discussion

Our results demonstrate that, with dynamic testing, there are differences in performance among shark teeth with different morphologies. Most notably, sixgill teeth did not cut as well as the other species (figure 4). Perhaps adult sixgills*,* which primarily eat teleosts and marine mammals [11] and do exhibit head-shaking behaviour [12], are eating prey whole and use their teeth to restrain rather than cut their prey. Tiger sharks feed on elasmobranchs, sea turtles, dugongs, teleosts, cephalopods and even sea birds [13–15], and silky and sandbar sharks feed primarily on teleosts, cephalopods, crustaceans and elasmobranchs [16–18]. These species cut their prey to pieces before eating it, and tiger sharks in particular are known to engage with very stiff prey tissues, like the carapace of sea turtles. Our apparatus could be used to develop a performance map of tooth shape cutting ability and prey type.

There is a trade-off between sharpness and wear resistance in man-made cutting tools—a finer edge is sharper but more easily folded over, chipped or rounded. Obsidian knives are wonderfully sharp, but are easily dulled, while the edge of a cleaver is robust but hardly sharp. Tiger and silky shark teeth showed rapid dulling after only a few interactions with prey tissues. As sharks continuously and rapidly replace their teeth, it is not surprising that their tools lie at the razor's edge of a sharp versus durable spectrum. The sixgill, which was less adept at cutting fish, also showed no signs of tooth dulling. Perhaps this reflects a lower rate of tooth replacement in this cold water species that probably has a much lower metabolic rate than carcharhinid sharks; both local water temperature and metabolic rate affect tooth replacement rate [19]. We predict there is a relationship between rapid dulling and frequent replacement. As suggested by our observed low rate of tooth chipping and by Whitenack *et al.* [20], it is likely that the selective pressure on tooth replacement is dulling rather than breakage.

Though our apparatus uses a saw to test shark teeth, a manufactured saw is a poor analogy for the sharp edge of a tooth. The teeth on saw blades are formed by removal of excess metal followed by a polishing or honing step. By contrast, shark teeth develop through deposition of material, with the outer enameloid layer reaching maximum thickness early in development [21]. The cross-section of a saw blade is either wedge shaped or hollow ground, but a shark tooth is a convex structure. It is difficult to see how we might manufacture a sharp edge based on the shape of a shark tooth, but performance testing of a biomimetic approach to edge design should be done in a dynamic setting.

Our dynamic testing device is more useful for measuring performance of teeth than traditional materials testing systems, because it tests draw and puncture performance at biologically relevant speeds. It also allows multiple teeth to interact with prey at the same time [22]. The reciprocating motion of the saw also produced bidirectional cutting, as seen in head-shaking behaviour. Our ability to test teeth over the course of many interactions with prey at biologically relevant strain rates allowed us to investigate wear and damage due to repeated use. Improvements to our design could include arrangement of teeth in a biologically relevant configuration, with tooth bases overlapping and tooth angles reflecting their position within the jaws. This device has great potential for further testing of teeth from other species, including model teeth from fossil taxa.

## Data Availability

Data for cutting ability and wear experiments are available for download at the Dryad Digital Repository: http://dx.doi.org/10.5061/dryad.92j80.
